# Toward nursing-integrated biomarker surveillance for immune-related adverse events during immune checkpoint inhibitor therapy

**DOI:** 10.3389/fmed.2026.1943642

**Published:** 2026-09-02

**Authors:** Jiafeng Jin, Liwen Zheng, Yali Tian, Minjuan Chu, Beinuo Cao

**Affiliations:** 1 Department of Nursing, Jiangsu Cancer Hospital & Jiangsu Institute of Cancer Research & The Affiliated Cancer Hospital of Nanjing Medical University, Nanjing, China; 2 Geriatric ICU, Jiangsu Province Hospital, Nanjing, China

**Keywords:** biomarkers, early warning, immune checkpoint inhibitors, immune-related adverse events, multidisciplinary care, oncology nursing, patient-reported outcomes, safety monitoring

## Abstract

Immune checkpoint inhibitors (ICIs) have transformed cancer care but can cause immune-related adverse events (irAEs) involving almost any organ system. Delayed recognition may permit reversible inflammation to progress to organ dysfunction, treatment interruption, prolonged immunosuppression, or death. Biomarker surveillance offers an objective complement to symptom assessment, yet no single marker is sufficiently accurate for universal prediction. This Mini Review examines the mechanistic basis and clinical evidence for nursing-led biomarker monitoring during ICI therapy. Targeted autoantibody, cytokine, or immune-cell assays may support selected clinical evaluations, whereas germline, T-cell receptor, and microbiome signatures remain investigational. Dynamic changes are generally more informative than isolated values, but interpretation is complicated by cancer burden, infection, concomitant treatment, and pre-existing disease. Nurses are positioned to connect serial laboratory trends with patient-reported symptoms, vital signs, treatment timing, and escalation pathways. Prospective studies are needed to validate multimarker panels and determine whether biomarker-informed nursing pathways reduce severe irAEs without unnecessary testing or interruption of effective immunotherapy.

## 1 Introduction

Immune checkpoint inhibitors targeting programmed cell death protein 1 (PD-1), programmed death ligand 1 (PD-L1), cytotoxic T-lymphocyte-associated protein 4 (CTLA-4), and related pathways have produced durable responses across many malignancies. Relief of inhibitory immune signaling can, however, disrupt peripheral tolerance and cause immune-related adverse events (irAEs). These toxicities may involve the skin, gastrointestinal tract, liver, endocrine glands, lungs, heart, kidneys, blood, muscles, or nervous system, and may arise during treatment or after treatment has ended (1–6).

International guidelines emphasize early recognition, organ-directed evaluation, timely grading, and coordinated multidisciplinary management (2–4). This makes surveillance a central component of immunotherapy delivery rather than an ancillary task. Nurses frequently obtain the first longitudinal evidence of change through treatment-cycle assessments, telephone calls, symptom reports, vital signs, and laboratory review. Their role is particularly important because early irAE symptoms—fatigue, diarrhea, cough, rash, weakness, headache, or appetite change—may be nonspecific and can be mistaken for cancer progression, infection, or treatment-independent comorbidity.

Biomarkers can provide objective evidence of immune activation or organ injury before overt clinical deterioration. In practice, the most useful candidates are not necessarily highly complex assays; routine blood counts, inflammatory indices, metabolic panels, thyroid function tests, glucose, creatinine, creatine kinase, and cardiac troponins are often more feasible for repeated monitoring. Advanced immune, genetic, and microbiome markers may improve biological understanding but remain less standardized. For implementation, biomarkers should be separated into three tiers: routine protocol-based tests; targeted clinician-directed assays for a suspected syndrome; and investigational immune, genetic, or microbiome assays that should remain within research protocols. This Mini Review focuses primarily on ICI-associated irAEs, for which the evidence base is most developed, and discusses how biomarker trends can be integrated into nursing-led early warning and safety management. Here, ‘nursing-led' refers to protocol-based symptom assessment, review of scheduled laboratory results, patient education, trend documentation, and escalation; it does not include independent diagnosis, immunotherapy interruption, or prescribing.

A targeted narrative search was conducted in PubMed/MEDLINE and Web of Science for English-language publications from January 2021 through 15 August 2026. Search terms combined “immune checkpoint inhibitor” or “cancer immunotherapy” with “immune-related adverse event”, “biomarker”, “blood test”, “eosinophil”, “nursing”, “patient-reported outcome”, “remote monitoring”, and organ-specific toxicity terms. Priority was given to current clinical guidelines, systematic reviews, larger clinical cohorts, implementation studies, and studies directly relevant to surveillance or escalation; reference lists were checked for additional eligible reports.

## 2 Mechanistic basis of irAEs and biomarker change

Checkpoint blockade restores antitumor T-cell activity, but the resulting immune activation is not confined to malignant tissue. Proposed mechanisms of irAEs include activation or expansion of autoreactive T cells, cross-reactivity between tumor and self-antigens, epitope spreading, B-cell activation and autoantibody production, cytokine imbalance, and direct effects on normal tissues expressing checkpoint molecules (1, 5, 7). These processes are not mutually exclusive and probably differ across organs and treatment regimens.

Biomarker changes reflect different levels of this pathophysiology. Systemic inflammation may increase C-reactive protein (CRP), interleukin-6 (IL-6), ferritin, or leukocyte-derived ratios. Immune-cell redistribution may alter lymphocyte subsets, eosinophils, or platelets. Autoantibodies and germline or T-cell receptor features may indicate susceptibility in selected patients, while organ injury releases tissue-linked markers such as alanine aminotransferase, creatine kinase, troponin, or thyroid hormones (8–13). The intestinal microbiota may also shape the balance between antitumor immunity and inflammatory toxicity; clinical studies and mechanistic work have linked microbial composition or microbial-directed immune responses to irAEs, especially colitis (14–16). Fecal microbiota transplantation has produced responses in selected refractory ICI-colitis cases, further supporting the biological relevance of this axis (16).

**Figure 1:**
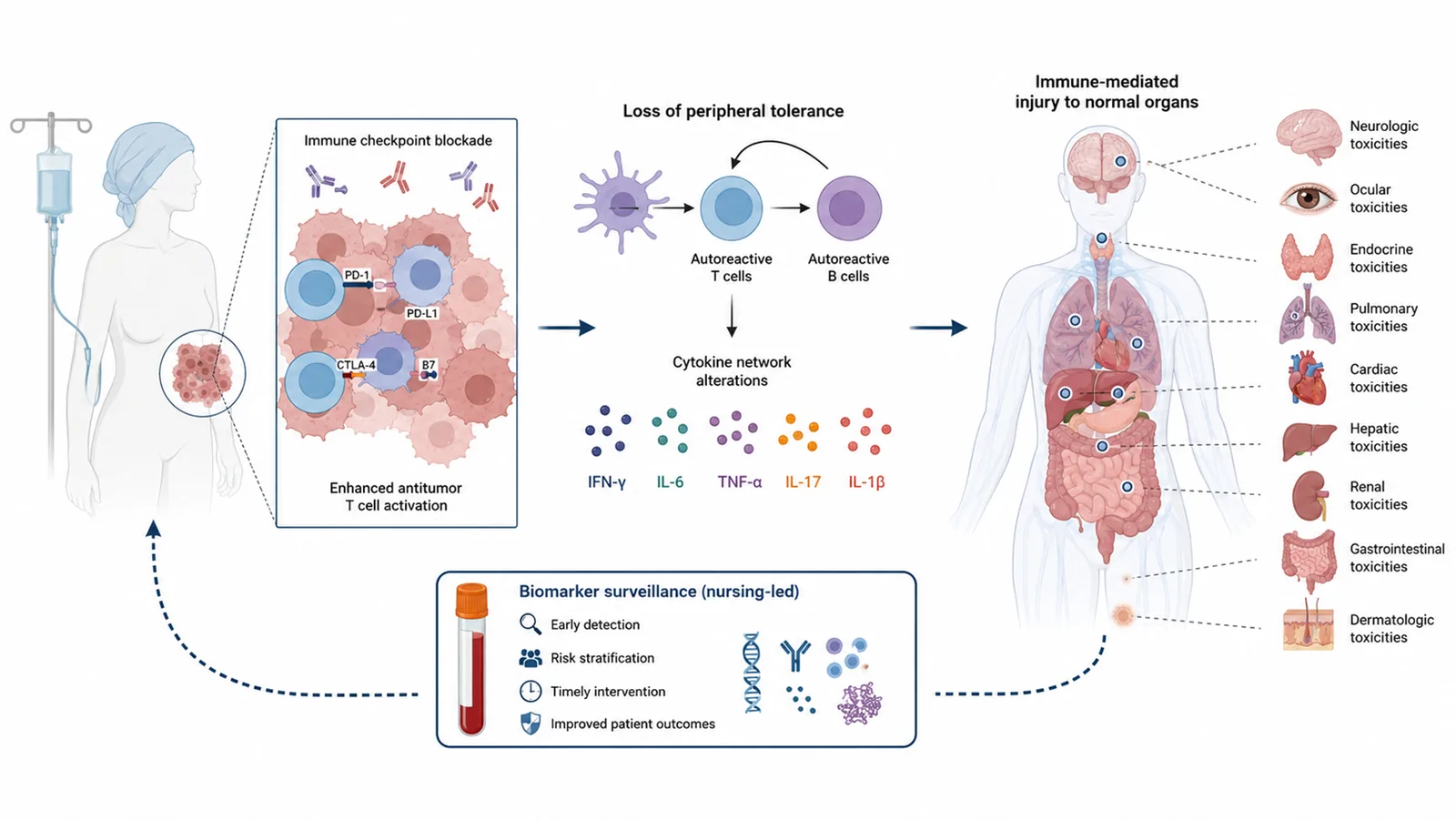
Mechanistic basis of nursing-led biomarker surveillance for immune-related adverse events during cancer immunotherapy. Immune checkpoint blockade enhances antitumor immunity but can disrupt peripheral tolerance, activate autoreactive T and B cells, alter cytokine networks, and injure normal organs. B7, B7 costimulatory molecule; CTLA-4, cytotoxic T-lymphocyte-associated protein 4; IFN-gamma, interferon gamma; IL, interleukin; PD-1, programmed cell death protein 1; PD-L1, programmed death ligand 1; TNF-alpha, tumor necrosis factor alpha.

The practical implication is that surveillance should not rely on a single universal biomarker. A biomarker may indicate general immune activation, tissue-specific damage, or a predisposing host state, and these meanings are not interchangeable. Serial patterns interpreted alongside symptoms and treatment timing are therefore more clinically informative than isolated abnormal results (17) (Figure 1).

## 3 Baseline assessment and accessible systemic biomarkers

A nursing-led monitoring plan begins before the first ICI dose. Baseline assessment should document autoimmune and endocrine history, cardiopulmonary symptoms, medications, recent infection, functional status, and pre-existing laboratory abnormalities. Guideline-informed baseline testing generally includes complete blood count, liver and renal profiles, glucose, and thyroid function, with additional endocrine, cardiac, or muscle testing determined by symptoms, comorbidity, regimen, and institutional policy (2–4). The purpose is not only prediction; baseline values allow subsequent changes to be distinguished from chronic abnormalities.

Complete blood count-derived markers are attractive because they are inexpensive and routinely available for longitudinal assessment. The neutrophil-to-lymphocyte ratio (NLR) has shown inconsistent directionality across studies. Lower baseline NLR has been associated with overall irAE occurrence in some cohorts, whereas rising NLR may accompany evolving organ inflammation and was reported before clinically apparent pneumonitis in one longitudinal analysis (18, 19). A large retrospective study also linked post-treatment NLR, baseline circulating tumor cell level, lactate dehydrogenase, and nutritional indices with irAE occurrence or severity (20). In the same retrospective study, post-treatment NLR showed poor discrimination (area under the curve 0.60; sensitivity 31%), underscoring that an associated signal may still be clinically inadequate as a stand-alone test (20). These findings support contextual trend review but do not establish a universal cutoff.

Low baseline NLR and a rising on-treatment NLR should not be treated as interchangeable or opposing thresholds, because they reflect different sampling times and potentially different biological states. No validated direction or universal cutoff currently supports using NLR alone to diagnose or grade an irAE. Nurses should instead verify timing and absolute cell counts, review infection, corticosteroid exposure, tumor burden, and concomitant treatment, and correlate the trend with symptoms and organ-specific tests; discordant signals should prompt clinician review rather than automatic action.

Eosinophils may provide another early signal. A multidisciplinary retrospective study found that an eosinophil proportion of at least 3% after two treatment courses was associated with subsequent irAEs across several tumor types and ICI regimens (21). Other studies have linked blood-cell changes and autoimmunity-related features to toxicity, but results vary by cancer, organ, and sampling time (22). A 2025 single-center retrospective cohort of 593 patients also associated a baseline eosinophil count above 250/µL and lower red-cell distribution width at baseline or early treatment with severe irAEs; the retrospective design and lack of external validation preclude use as a stand-alone screening rule (23). CRP, albumin, lactate dehydrogenase, platelet-to-lymphocyte ratio, systemic immune-inflammation index, and prognostic nutritional index are similarly accessible but nonspecific. They may be most useful when interpreted as within-patient changes rather than stand-alone diagnostic tests.

Inflammatory markers can also help characterize established toxicity. In patients with ICI-associated cardiotoxicity, elevations in cardiac biomarkers and inflammatory indices were observed at toxicity onset, and a higher neutrophil-to-eosinophil ratio was associated with more severe events and mortality (24). Such data illustrate the potential value of combined organ-specific and systemic signals, while also showing why biomarker interpretation should trigger clinical evaluation rather than automatic attribution to an irAE.

## 4 Immune, genetic, and microbiome biomarkers: promise and limitations

Autoantibodies are biologically plausible predictors because checkpoint blockade may unmask latent autoimmunity. Systematic reviews and cohort studies have reported associations between selected baseline or emergent autoantibodies and organ-specific irAEs, but associations are heterogeneous and sometimes counterintuitive (9–11). Routine broad autoantibody screening is therefore not supported for every patient. Targeted testing is more appropriate when symptoms suggest a rheumatic, neurologic, endocrine, or neuromuscular syndrome.

Host genetics and immune repertoire features may eventually support individualized risk estimation. Germline variants have been associated with anti-PD-1/PD-L1 toxicity, and a T-cell receptor beta variable gene polymorphism was reported to predict irAEs in checkpoint blockade cohorts (12, 13). These observations remain exploratory because replication, ancestry diversity, assay availability, and clinical thresholds are limited.

As discussed in Section 2, intestinal microbiome profiles may influence irAE susceptibility, particularly colitis. For surveillance, however, clinical translation is limited by diet, antibiotics, geography, sequencing platforms, cancer type, and sample handling. No reproducible signature or threshold currently supports routine testing; microbiome assays should therefore remain investigational.

A further clinical controversy is that irAEs are often associated with favorable ICI outcomes. A meta-analysis of randomized studies supported an association between irAEs and treatment efficacy (25). This relationship should not be interpreted as a reason to tolerate progressing toxicity or to use toxicity as a desired surrogate. The nursing objective remains early detection and organ preservation, with treatment decisions made by the multidisciplinary team.

## 5 Organ-specific biomarker surveillance

Organ-specific surveillance should be symptom-informed and protocolized. Gastrointestinal irAEs may begin with increased stool frequency, abdominal pain, blood, or mucus. Stool infection testing remains essential, and fecal inflammatory markers can support evaluation in selected settings. Tissue studies show that activated interferon-gamma-producing CD8-positive tissue-resident memory T cells are prominent in ICI colitis, but such profiling is not a routine monitoring tool (26). Reviews and guidelines support prompt assessment because diarrhea severity may underestimate mucosal inflammation (27, 28). Liver toxicity requires serial alanine aminotransferase, aspartate aminotransferase, alkaline phosphatase, and bilirubin interpreted with medication, infection, obstruction, and metastatic disease (28, 29).

Endocrine irAEs often present with nonspecific fatigue, nausea, headache, dizziness, thirst, or altered mental status. Serial thyroid-stimulating hormone and free thyroxine are among the most established monitoring tests. Glucose should be reviewed regularly, and morning cortisol, adrenocorticotropic hormone, electrolytes, or pituitary testing should be obtained when symptoms suggest adrenal or pituitary dysfunction (30). Real-world data on ICI-induced diabetes show that inadequate cycle-by-cycle glucose monitoring can delay diagnosis and increase the risk of diabetic ketoacidosis (31). Because endocrine injury may be irreversible, nursing education should emphasize symptom reporting even when routine laboratory results were previously normal.

Cardiac and neuromuscular toxicities are uncommon but potentially rapidly fatal. Risk-adapted surveillance may include electrocardiography and cardiac troponins, particularly early in treatment or with combination regimens, according to local cardio-oncology protocols (32). New chest pain, dyspnea, palpitations, syncope, ptosis, dysphagia, or proximal weakness should trigger urgent assessment with troponin, creatine kinase, electrocardiography, and specialist evaluation. Cardiac biomarkers should be interpreted together with symptoms, imaging, and alternative causes; they are not diagnostic in isolation (24, 32, 33).

Skin toxicity is often visible before laboratory changes, but eosinophilia and inflammatory signatures may accompany selected phenotypes. Mechanistic work indicates distinct interferon- and type 2-associated pathways across cutaneous irAEs (34). Renal surveillance relies on creatinine trends, urinalysis, hydration status, and medication review. Hematologic irAEs require attention to falling hemoglobin, neutrophils, or platelets and may need urgent specialist input because evidence for management remains limited (35). Across organ systems, the nurse's task is to recognize discordance—such as mild symptoms with rapidly changing biomarkers, or severe symptoms despite apparently normal routine tests—and escalate accordingly.

## 6 Nursing-led integration of symptoms and biomarker trends

Nursing-led monitoring does not mean independent diagnosis or treatment. It describes a structured role in longitudinal assessment, patient education, laboratory review, communication, and escalation within agreed multidisciplinary pathways. Evidence for biomarker associations is distinct from evidence supporting nurse-led or electronic patient-reported outcome interventions; their integration into one biomarker-informed nursing pathway has not been prospectively validated. We therefore propose an unvalidated four-component model. Nurses may conduct protocol-authorized symptom assessments, review scheduled laboratory results, confirm and document trends, and initiate predefined communication or escalation steps. Ordering diagnostic investigations outside standing protocols, assigning the diagnosis or Common Terminology Criteria for Adverse Events (CTCAE) grade of an irAE, interrupting or resuming immunotherapy, and prescribing corticosteroids or other immunosuppressive therapy remain medical decisions. The four-part framework and its safeguards are operationalized in Table 1.

**Table 1:** Proposed operational framework and professional boundaries for nursing-integrated irAE biomarker surveillance.

Component	Protocol-based nursing actions	Decisions reserved for clinicians	Safety safeguards
Baseline preparation	Document history, medications, symptoms, and baseline values; review scheduled CBC, liver/renal, glucose, and thyroid tests; provide contact instructions.	Define individualized additional testing and determine treatment eligibility.	Record chronic abnormalities and avoid indiscriminate expanded panels.
Active-cycle assessment	Use structured symptom and vital-sign review; compare scheduled laboratory results with baseline and prior cycles.	Order organ-specific diagnostics and adjudicate cause and CTCAE grade.	Check timing, infection, concomitant therapy, sample quality, and trend direction.
Escalation rules	Confirm symptoms, follow standing repeat-test pathways, document changes, and notify the clinician promptly.	Decide immunotherapy interruption/resumption and prescribe corticosteroid or other immunosuppression.	Do not act on one isolated nonspecific result; urgent symptoms or critical values require immediate review.
Remote monitoring	Review ePRO or telephone alerts, contact the patient, and close the communication loop.	Set alert thresholds, response times, coverage, and diagnostic or treatment actions.	Calibrate alert burden, protect privacy, and retain accessible non-digital pathways.

This proposed framework has not been prospectively validated and may require adaptation to local staffing, scope of practice, laboratory access, treatment protocols, and escalation resources. CBC, complete blood count; CTCAE, Common Terminology Criteria for Adverse Events; ePRO, electronic patient-reported outcome; ICI, immune checkpoint inhibitor; irAE, immune-related adverse event.

First, baseline preparation should include a symptom inventory, comorbidity and medication review, documentation of baseline laboratory values, and clear education about delayed and multisystem toxicities. Patients and caregivers should receive plain-language instructions on when and how to contact the team. A nursing study of severe irAEs in lung cancer identified delays between symptom onset and hospital presentation and highlighted the need for more specific education and telephone access (36).

Second, each treatment cycle should include active symptom inquiry rather than reliance on spontaneous reporting. The assessment should cover bowel habit, cough and exertional dyspnea, rash and pruritus, fatigue and weakness, headache or visual change, thirst or polyuria, chest symptoms, and neurologic complaints. Laboratory values should be compared with the patient's baseline and prior cycle, not only with population reference ranges. A small but consistent change across several cycles may be more informative than one isolated abnormal value.

Third, abnormal findings should be linked to predefined escalation rules. A rising aminotransferase level, new creatinine increase, falling free thyroxine, elevated troponin, or progressive eosinophilia should prompt symptom confirmation, medication and infection review, repeat or organ-specific testing, and timely clinician notification. Nurse-led telephone triage and multidisciplinary immunotherapy toxicity programs have demonstrated the feasibility of structured toxicity assessment and may reduce avoidable hospital use or support earlier intervention (37, 38).

Fourth, remote monitoring can extend surveillance between visits. A randomized immunotherapy trial found that electronic patient-reported outcome (ePRO) follow-up reduced grade 3–4 irAEs, emergency department visits, and treatment discontinuation compared with traditional follow-up (39). Systematic review evidence supports the feasibility and acceptability of ePRO monitoring, although outcome evidence remains limited (40). eHealth models commonly route worsening symptoms to triage nurses, who contact patients and coordinate management (41). Co-design studies emphasize the need for comprehensible questions, actionable advice, alignment with clinical workflow, 24-hour contingency plans, and clear responsibility for alerts (42). Broader oncology trials also show that electronic symptom monitoring can improve symptom control and quality of life (43), while daily remote monitoring studies identify usability, adherence, and clinician engagement as important implementation variables (44).

Digital systems should supplement, not replace, accessible telephone and face-to-face pathways. Older adults, patients with low digital literacy, and those with language or socioeconomic barriers may otherwise be excluded. Alert fatigue is another risk; biomarker and symptom thresholds should be calibrated so that urgent signals are visible without creating unsustainable workload.

## 7 Discussion and future directions

Biomarker-guided irAE surveillance is clinically attractive because it seeks to translate immune dysregulation into measurable signals. Its proposed near-term role is to evaluate whether routinely available tests can complement structured nursing assessment, not to imply that a highly specific predictive assay or a validated integrated pathway already exists. Complete blood count trends, CRP, metabolic panels, thyroid function, glucose, creatinine, creatine kinase, and troponin are inexpensive and repeatable, but each is affected by cancer burden, infection, concomitant chemotherapy, nutritional status, and chronic disease. Their value therefore depends on context and serial interpretation.

Expanding panel size or testing frequency is not inherently safer. Nonspecific abnormalities can generate false-positive alerts or incidental findings, leading to repeat sampling, unnecessary workups, patient anxiety, financial burden, and avoidable interruption of effective immunotherapy. Monitoring should therefore be risk-adapted and protocol-defined. Unless a critical value or concerning symptom requires urgent review, an isolated low-grade deviation should be checked against the pretreatment baseline, symptoms, sample quality, infection, concomitant medicines, and trend direction before further escalation.

The strongest nursing contribution is integration. Nurses can connect symptom timing, treatment exposure, patient-reported changes, vital signs, and biomarker kinetics, then activate organ-specific pathways. This longitudinal perspective is difficult to reproduce with a single clinic visit. It also supports continuity after ICI discontinuation, when delayed irAEs may still occur. Structured documentation should record baseline status, trend direction, patient education, actions taken, and response to intervention.

Several gaps limit implementation. Most candidate biomarkers were identified retrospectively, with heterogeneous definitions of irAEs and different sampling schedules. Many studies combine tumor types and ICI regimens, while organ-specific events are relatively uncommon. Multimarker panels may improve prediction but risk overfitting and may not generalize across populations. A 2026 SEER-Medicare study of 2,729 older adults with advanced non-small cell lung cancer reported an internally validated severe-irAE risk model (bootstrap-corrected area under the curve 0.85), but its claims-based endpoint systemic immunosuppression plus ICI delay or discontinuation does not correspond directly to CTCAE grade 3 or higher, and external validation is still required (45). Prospective studies should compare predefined nursing-led pathways with usual care and evaluate clinically meaningful outcomes: time from symptom onset to recognition, peak irAE grade, hospitalization, organ recovery, immunosuppression exposure, treatment interruption, quality of life, and workload.

Future studies should evaluate adaptive rather than fixed monitoring intensity. Baseline autoimmune disease, combination ICI therapy, early biomarker shifts, and previous irAEs are candidate—not validated—factors for determining monitoring intensity. Algorithms should display trends rather than isolated values and should integrate ePROs with laboratory and medication data. Importantly, automated alerts require human oversight, transparent thresholds, and equity-sensitive alternatives.

In conclusion, available evidence supports continued evaluation—not clinical validation—of nursing-integrated biomarker surveillance during immune checkpoint inhibitor therapy. Biomarker association studies and nursing or electronic patient-reported outcome intervention studies provide different forms of evidence, and no biomarker currently replaces clinical assessment or independently guides diagnosis, CTCAE grading, treatment interruption, or immunosuppression. The proposed layered framework combines baseline characterization, serial guideline-recommended safety tests, symptom-triggered organ biomarkers, patient-reported outcomes, and protocol-based escalation. Its safety, workload, equity, generalizability, and patient-outcome effects require prospective validation before routine international implementation.
